# Supplementary material for: Frequency of non-communicable diseases in people 50 years of age and older receiving HIV care in Latin America
Source: PLoS One. 2020 Jun 17;15(6):e0233965. doi: 10.1371/journal.pone.0233965 (PMC7299309; doi:10.1371/journal.pone.0233965)
Supplement: Attachment — Submitted filename: Reviewers Comments.docx [file pone.0233965.s001.docx]

This paper aims to estimate the prevalence of NCDs amongst PLHIV in Latin America using CCASAnet cohort data. This is a clear, well written and timely study which adds important data to a field that is gaining increasing momentum. Particular value is the region these findings are reporting on, with Latin America being a region were such data is lacking compared to other regions such as North America and Europe and even SSA.

My main concern centre around the choice of sub-analysis and its focus on age at enrolment only instead of maximising use of this large dataset to e.g. generate regression analysis looking at risk factors for NCDs. See detailed comments below

Detailed comments:

- It is important to distinguish the impact of aging PLHIV on HIV care and aging in general on the health system (including primary care). HIV remains a low prevalent disease and thus is unlikely to have a marked impact on health systems more generally compared to the aging general population. The abstract and introduction should be updated accordingly.
- Stratifying by age at enrolment seems an odd choice of sub-analysis as a simple descriptive overall analysis would already provide important results. As stated by authors in introduction ART exposure, previous health history, HIV duration and severity could drive NCD burden beyond age and may drive different NCDs. A sub-analysis by age at enrolment is not adequate to control for these factors, as hinted in introduction. I assume the CCASAnet cohort provides data on some of these factors and other (e.g. smoking, BMI, etc). Would the authors not consider dissecting this issue of NCD further by looking at risk factors for NCDs as other studies from HIC have done using regression to tease out effect better? This seems to be a major limitation of the paper and such addition would provide a wealth of additional data to this paper. If this is not feasible this would fully discussed in discussion
- Authors should provide a little more information on the CCASAnet cohort for the reader. Including that it compromises clinical data from 7 countries, how NCDs are defined (e.g. are clinical definitions the same across facilities, if so provide these (e.g. is hypertension 140/90 or 130/80?), if not this should be stated and mentioned in discussion as limitation)
- Authors should explain why their analysis is limited to data from only 5 countries and not all 7 countries in CCASAnet. I assume this is because some facilities did not report on NCDs?
- How was enrolment defined? HIV diagnosis? At ART initiation? Other?
- NCD prevalence is higher in <50 but by end of follow-up it was the same. Authors should dissect this somehow to better help the reader understand this difference at the beginning. Authors should hint at an explanation in result section and elaborate in discussion. The same comment applies when reporting on individual NCDs in the next section. Why dyslipidaemia for example is 27% in <50s and 3% in over 50s).
- As pointed out the lower prevalence in this study compared to other settings may be explained by lower screening? What do guidelines say about frequency of screening in PLHIV, is there data on gap between guidelines and real world (e.g. as done in <https://www.ncbi.nlm.nih.gov/pubmed/28578613> for the Netherlands)? Could they also provide data on eg prevalence of hypertension in HIV-negative from both regions to support the hypothesis of genetic/risk factor does result in lower prevalence in Latin America?
- The discussion is somewhat short. Authors should elaborate on a number of points:
  - How do the results compare to other data from the region and beyond show eg the Brazil and Mexico data mentioned in intro
  - What are the implications for clinical care (prevention, screening, guidelines, polypharmacy eg Smit et al Lancet ID 2015)
  - What gaps remain (e.g. more information on proportion of those screened who are treated and controlled) and what data would need to be collected. This is a great opportunity to highlight not just a huge burden but also call for more research – use it ☺
  - How generalisable are the results given that it includes only 5 countries from the region. E.g. how different are some regions in Latin America.
  - How much data do Mexico and Brazil contribute to CCASAnet data (not the main contributors when looking at numbers overall but not sure for >50yo). As the previous study into NCD from the region included data from Mexico and Brazil it would be good for authors to clearly highlighted added benefits of this study in discussion in relation to existing evidence (multi-cohort, larger number, more generalisable to whole region etc).
  - Would it be useful to have cohorts with controls as in US and Europe to better understand the excess risk of HIV disease and ART on NCDs

General comments

- Authors could use epidemiological terminology e.g prevalence instead of frequency, proportion etc
- When reporting on which NCDs contribute the largest burden, authors could focus on reporting the 3 main NCDs. The number of ‘main’ NCDs reported varies between abstract, results and discussion.
- For figures authors may consider using increasingly darker shades from 0 to multimorbidity instead of a darker colour in the middle for 1NCD? If staying in grey shades and increasing in darkness makes it tricky to read consider using patterns (e.g. no pattern=0 NCDs, light pattern is 1 and dark pattern is 2+)

Minor comments

- In abstract could authors be more explicit in abstract that NCD prevalence was estimated amongst PLHIV (not general population attending centres)
- In conclusions of abstract authors may also like to elude to the fact that the burden seems to be increasing over time
- In introduction could authors state that the study was “from Name et al” instead of “from our colleagues” colleagues?
- In introduction when reporting on reasons for excess mortality authors should say “possible reason could be the increased presence” instead of “is”
- Discussion could benefit from use of paragraph to distinguish individual sections.
- In discussion authors state that HTN, dyslipidaemia, psychiatric disorders AND diabetes are the main NCDs but diabetes was not mentioned in result or abstract.
- It would strengthen discussion to mention what drives observed trends when discussion difference in NCD burden at enrolment and end of follow-up (higher incidence)
- I am not sure I understand the sentence “Another potential limitation is that the prevalence …”
- The sentence “As such, we focus… “is misleading given the point on underdiagnosis. Authors may want instead to comment on gaps could be addressed for future analysis (analysis of screening practice to see if it is true underdiagnosis, that underscreening compared to guidelines has also been reported in HIC eg <https://www.ncbi.nlm.nih.gov/pubmed/28578613>.
