# Supplementary material for: Frequency of non-communicable diseases in people 50 years of age and older receiving HIV care in Latin America
Source: PLoS One. 2020 Jun 17;15(6):e0233965. doi: 10.1371/journal.pone.0233965 (PMC7299309; doi:10.1371/journal.pone.0233965)
Supplement: Attachment — Submitted filename: Response to reviewers_20200119.docx [file pone.0233965.s002.docx]

Mexico City, January 20^th^, 2020

**Bruno Pereira Nunes, Ph.D.
Academic Editor
PLOS ONE**

Dear Dr Pereira.

We appreciate that a revised version of our manuscript is being considered for publication at PLOS ONE. We also appreciate the thorough review and thoughtful comments by our peers. We are submitting a revised version of the manuscript that intends to address the points raised during the review process. In the next pages of this document we provide a response to each point, indicating the corresponding changes in the manuscript. We are also submitting two versions of the revised manuscript: in one version we highlighted all changes we did to our originally submission and a second version with all changes accepted.

**Responses to Reviewers:**

**Reviewer #1:**

Non-communicable diseases and the accumulation of them within PLWH is of significant and growing concern given an expected increase in the number of PLWH aged >50 years of age. As authors note, there are a limited number of studies quantifying the epidemiology of NCDs and multimorbidity in a Latin American setting, particularly in large numbers. This important study provides evidence of a growing prevalence of NCDs, but would be strengthened by refining study objectives, clarifying methods and implications of findings.

**Major Comments**
1. Authors conclude that regardless of age at enrollment, study findings suggest that a high prevalence of NCDs and multimorbidity were observed. How are these results reconciled with their statement that planning and provision of complex, primary care for PLWH should target mainly those who are 50+?

***ANSWER:*** *All patients included in the study were 50 years and older at the start of observation time for this study. We limited application of conclusions from the study are to people older than 50yo.*

*We made changes at the Introduction and Methods sections that we think will help to better understand the purpose this analysis. Our overall goal was to provide further information on the frequency of comorbidities in people ≥50 years of age receiving care for HIV in our region, and contribute to a better understanding of the magnitude of this problem. This information might be used to inform planning care, and estimate costs and needs of HIV-related healthcare in Latin America, now that the population of people living with HIV is aging.*

2. To be included in this observational cohort analysis, patients being observed for clinical care **need to contribute at least 2 clinical visits, at least one of which occurred during the study period of 2000-2015 (line 103).** How are new or “incident” events determined if only one visit was available? Did this analysis take place within a subset of the full study population of n=3415?

***ANSWER:*** *Per inclusion criteria, there were no patients with only one visit available. Patients were included if they had at least 2 clinical visits to ensure all patients contributed observation time. Patients with only one clinic visit were excluded. We performed three separate analysis; inclusion criteria were identical for the whole study population (*n=3,415)*.*

3. In the discussion, authors note that a limitation of their study is that events may have been underascertained. Given the inclusion of multiple study sites and the potential for heterogenous data collection (ie the point in time during which data was collected may vary by site), did authors have a systematic way of ensuring that all constituent NCDs had data available during overlapping periods of time? For example, to assess whether a person had X of 8 proposed categories of NCDs, presumably all 8 categories of NCDs were being collected at the same time.

**ANSWER:** *All 8 categories of NCDs were being collected at the same time in all sites during the whole study period, per cohort design (see McGowan CC, et al. Int J Epidemiol. 2007; reference 19). The cohort has standardized data collection procedures and quality assurance methods. We added a description of these in the subsection “Cohort setting and procedures” and references (pages 5-6, lines 116-143; references 19-26) in the new version of the manuscript. However, screening for NCDs is not standardized across all clinical sites and might have varied by center over time. We have added this acknowledgement to the limitations in the Discussion section (page 19 lines 384-391). We also expanded the discussion of this potential limitation, adding references that support the systematic under-ascertainment of NCDs in adults receiving care for HIV during routine care (references #25, #26 and #39).*

4. As visit structure of participants is driven by clinical care seeking behavior, “incident” NCDs may not represent true biological onset and should be addressed in the limitations. Relatedly, as in line 156, prevalent cases were removed at or before the start of follow-up. It would be helpful to clarify whether there was window of time during which events were considered “prevalent” (for example, 6 months before and after enrollment).

**ANSWER:** *We agree with the reviewer’s observation that health care seeking behavior affects recognition and capture of diagnoses for these conditions. We acknowledged this in the Discussion (see page 19, lines 388-391). We don’t know the exact biologic onset of many NCDs (cancers, diabetes, heart disease, etc.) and clinically we use date of diagnosis as disease onset, recognizing this limitation in medicine. Using date of diagnosis as event is consistent with clinical practice.*

*Prevalent comorbidities included diagnoses recorded before or at the start of observation time (first clinic visit for those ≥50 years old at entry or first visit after age 50 for those <50 years old at entry). Incident diagnoses were all non-repeating diagnoses recorded after start of observation period until death, loss to follow-up, or administrative censoring. We have clarified these definitions in the Methods section (see page 8, lines 185-189).*

5. In line 231, authors discuss results presented in Table 2. More specifically, that incidence rates of NCDs were higher among PLWH who enrolled in care when they were >50 years old, as compared with individuals who were <50 years old at enrollment. Presumably, to adjust for age “patients <50 at enrollment started follow-up at their first visit after their 50th birthday” (line 146-47), however, are the groups comparable if those <50 years at enrollment may have experienced incident NCDs at an earlier age during the course of their clinical care, simply not captured by the study period?

***ANSWER:*** *In our view, these two groups are not comparable. The reason for presenting the groups by age at enrolment is not to attempt to adjust for age, which would not be appropriate through stratification. Our purpose was to describe separately two groups with distinctive clinical histories, so we did no formal comparison between groups.*

*In this new version, we modified the text and avoid statements suggesting that we are comparing incidence of diseases between these groups. We also modified the language in the Methods (See page 7, lines 163-166), Results (see page 16, lines 328-331) and Discussion (see page 16, lines 315-320, page 17, 336-339) to address this comment.*

*We did substantial modifications to the Methods section, to address this and other comments by both reviewers. This including extensive re-writing to provide clearer and more explicit definitions. We did not include in this response all changes to this letter of response, but reviewers will be able to track them easily as all changes are identified with the track-changes function in Word. We hope that the new version reflects more accurately our thinking during the analysis*.

*Finally, had those <50 years at enrollment been diagnosed with NCDs at an earlier age during the course of their medical care, these were recorded as “prevalent” non-communicable disorders at cohort entry.*

6. The denominator for prevalence was “all older patients older than 50 with at least 1 visit after July 15th”. Please provide rationale.

***ANSWER:*** *In the analysis to estimate the annual prevalence of comorbidities (summarized in Figure 1), patients were included if had at least a clinical visit after July 15^th^ in the year analysed to ensure they were retained and actively receiving care that year. We modified the description of the analysis to provide a clearer description of this selection (page 9, lines 213-217).*

7. In line 155, is “start and end of follow-up” all time after 50 years of age up until death/NCD event/2015?. Similarly, in line 161, is “total time of follow-up” = end of follow up – start of follow up?

***ANSWER:*** *This is correct.*

**Minor Comments**
1. The abstract’s background reads as though authors intended to quantify frequency of NCDs, individual and in aggregate, among PLWH older than 50. However, it seems their objective to describe the rate at which they occur (incidence) was excluded.

***ANSWER:*** *Thank you for this observation. We have corrected the Abstract's background to include incidence (page 2, lines 42-45).*

2. In Line 83, “multimorbidity was the norm in both centers”. Please quantify and cite.

***ANSWER:*** *We modified this sentence as recommended. The text in the new version states:* “*Moreover, the mean number of comorbidities was 1.4 in Mexico and 2 in Brazil, with an overall prevalence of multimorbidity of 63% in Brazil [15,16].”.* *References have been added (page 4, lines 86-88, References #15 and #16)*

3. The outcomes of NCDs appear to be a blend of “registered” and “reported” events. Is this distinction referring to whether outcomes were self-reported vs physician diagnosed?

***ANSWER:*** *We used the terms “registered” and “reported” for any event in the dataset without distinction. In the revised version, we consistently use the term “registered” for all events to avoid confusion. Each center collect data during routine HIV-care and might register a new non-communicable disease if this was diagnosed in the center since their last HIV-care visit, if the patient self-reported to have been diagnosed with a new NCDs since their last HIV-care visit elsewhere or if in that visit, their HIV-care physician diagnosed a new NCD. This data is collected from the clinical files and medical notes.*

4. Please provide citations for the statement in line 270 “differences in the frequency of environmental, genetic, and individual risk factors for NCDs might explain disparities”.

***ANSWER:*** *We added the references #32 and #33.* *Please see page 17, lines 344-346.*

5. Statistical tests in Table 1 were performed, but not described in the Methods’ statistical analysis plan.

***ANSWER:*** *Thanks for pointing this out. We added the description in the methods section (page 9, lines 201-204), as follows:*

*“We compared sociodemographic, clinical and medical history characteristics between these groups to verify and document our assumption that they are different using chi-squared tests for categorical variables and Kruskal-Wallis for continuous variables.”*

**Reviewer # 2**

This paper aims to estimate the prevalence of NCDs amongst PLHIV in Latin America using CCASAnet cohort data. This is a clear, well written and timely study which adds important data to a field that is gaining increasing momentum. Particular value is the region these findings are reporting on, with Latin America being a region were such data is lacking compared to other regions such as North America and Europe and even SSA.

My main concern centre around the choice of sub-analysis and its focus on age at enrolment only instead of maximising use of this large dataset to e.g. generate regression analysis looking at risk factors for NCDs. See detailed comments below

Detailed comments:

- It is important to distinguish the impact of aging PLHIV on HIV care and aging in general on the health system (including primary care). HIV remains a low prevalent disease and thus is unlikely to have a marked impact on health systems more generally compared to the aging general population. The abstract and introduction should be updated accordingly.

***ANSWER:*** *We completely agree with this comment. We modified the Abstract and the main text Background sections to accurately reflect this. The new version states: “A growing population of older adults with HIV will increase demands on HIV-related healthcare.” (page 2, lines 39-40) and “As the population of PLWHIV ages, co-morbidities resulting from natural aging, effects of chronic inflammation, and long-term use of cART are expected to also grow, increasing demands on HIV-related health care [5,6].” (page 3, lines 68-71).*

- Stratifying by age at enrolment seems an odd choice of sub-analysis as a simple descriptive overall analysis would already provide important results. As stated by authors in introduction ART exposure, previous health history, HIV duration and severity could drive NCD burden beyond age and may drive different NCDs. A sub-analysis by age at enrolment is not adequate to control for these factors, as hinted in introduction.

***ANSWER:*** *We agree that stratifying by age at enrollment does not adequately control for differences driving NCD in these two groups. In our view, these two groups are not comparable. We elaborate further in response to commentary #5 of reviewer 1. Briefly, we are presenting the groups by age at enrolment to describe separately two groups of our interest that are likely not part of the same population, and there is no simple method to adjust for their differences. We acknowledge the language we used in the abstract and main text does not conveys our reasoning clearly, so we modified different sections throughout the manuscript for clarity purposes (see page 7 lines 163-166, page 16, lines 328-331; and page 16, lines 315-320 and page 17, 336-339 in the Methods, Results and Discussion sections)*

- I assume the CCASAnet cohort provides data on some of these factors and other (e.g. smoking, BMI, etc). Would the authors not consider dissecting this issue of NCD further by looking at risk factors for NCDs as other studies from HIC have done using regression to tease out effect better? This seems to be a major limitation of the paper and such addition would provide a wealth of additional data to this paper. If this is not feasible this would fully discussed in discussion.

***ANSWER:*** *CCASAnet cohort does not routinely collect data on some of these other primary risk factors for NCDs (e.g., smoking). The goal of this paper was to focus on the growing population of PLWH ≥50 years and the burden of their other medical comorbidities. Evaluation of NCD risk factors is the next step to identify who is at greatest risk and identify modifiable risk factors for the development of NCDs (regardless of age) in the region. An important but different question that we agree needs further research. We have added some sentences related to this in the discussion, please see page 18, lines 376-377 and page 19, lines 381-384.*

- Authors should provide a little more information on the CCASAnet cohort for the reader. Including that it compromises clinical data from 7 countries, how NCDs are defined (e.g. are clinical definitions the same across facilities, if so provide these (e.g. is hypertension 140/90 or 130/80?), if not this should be stated and mentioned in discussion as limitation)

***ANSWER:*** *We modified substantially the Methods section to address this and other comments by reviewers. In the new version, we included a new sub-heading titled: “Cohort settings and procedures” where we describe how data on NCD is defined and collected (pages 5-6, see lines 116-143). In the new version, we also address the limitations of our definitions and collection methods (see pages 18-19, lines 370-395).*

- Authors should explain why their analysis is limited to data from only 5 countries and not all 7 countries in CCASAnet. I assume this is because some facilities did not report on NCDs?

***ANSWER:*** *This is correct. We included data from sites where information on non-communicable disorders is available (6 centers in 5 countries). We added in the Methods section (see pages 6-7, lines 147-152) a statement to clarify this point: “We included data of patients receiving care at the six centers where data on non-communicable diseases are collected (Fundación Huesped in Argentina, Instituto Nacional de Infectologia Evandro Chagas -Fiocruz in Brazil, Fundación Arriarán in Chile, Instituto Hondureño de Seguridad Social and Hospital Escuela Universitario in Honduras, and Instituto Nacional de Ciencias Médicas y Nutrición Salvador Zubirán in Mexico).”*

- How was enrolment defined? HIV diagnosis? At ART initiation? Other?

***ANSWER:*** *CCASAnet have standardized definitions for these variables. Details and definitions are described in reference #19 and the set of variables are publicly available in documents accessible through: https://www.ccasanet.org*

*For the reviewer, definitions according to these documents are:*

*Enrollment: Date of enrolment in HIV care in each site.*

*HIV diagnosis: Date of first HIV positive test.*

*ART initiation: date of first ART regimen.*

NCD prevalence is higher in <50 but by end of follow-up it was the same. Authors should dissect this somehow to better help the reader understand this difference at the beginning. Authors should hint at an explanation in result section and elaborate in discussion. The same comment applies when reporting on individual NCDs in the next section. Why dyslipidaemia for example is 27% in <50s and 3% in over 50s).

***ANSWER:*** *We have added some sentences to explain our interest to describe separately two groups with distinctive clinical histories and the potential reasons of those differences. Please see our response for the comment 5 of reviewer 1 and Methods (pages 7, lines 163-166) and Results section (page 16, lines 328-331).*

*In brief, we attribute differences in healthcare seeking behaviors and access as the main contributor to differences in baseline prevalence of any NCDs and multimorbidity. After enrollment in care, we suspect that greater opportunities of screening and diagnosis of NCDs during HIV care and dyslipidemia after ART initiation as the main explanations for apparent differences in incidence after enrollment in care in people enrolled later in life (>50yo).*

- As pointed out the lower prevalence in this study compared to other settings may be explained by lower screening? What do guidelines say about frequency of screening in PLHIV, is there data on gap between guidelines and real world (e.g. as done in <https://www.ncbi.nlm.nih.gov/pubmed/28578613> for the Netherlands)? Could they also provide data on eg prevalence of hypertension in HIV-negative from both regions to support the hypothesis of genetic/risk factor does result in lower prevalence in Latin America?

***ANSWER:*** *There is a lack of guidelines for prevention, screening, care and treatment for specific NCDs for the general population at the country level, not to mention guidelines for HIV care for people 50 years and older (page 19, lines 391-395, references #44 to #46 ). We agree that lower screening for non-communicable diseases could be a reason for lower estimates of prevalence in our study population. Nonetheless, there is little information on the gaps between recommendations/guidelines and practice, as we indicate in this new version with added sentences in this section. We address these issues in the Discussion of this revised version (Page 19, lines 391-395, references #40 to #42).*

- The discussion is somewhat short. Authors should elaborate on a number of points:
  - How do the results compare to other data from the region and beyond show eg the Brazil and Mexico data mentioned in intro
  - What are the implications for clinical care (prevention, screening, guidelines, polypharmacy eg Smit et al Lancet ID 2015)
  - What gaps remain (e.g. more information on proportion of those screened who are treated and controlled) and what data would need to be collected. This is a great opportunity to highlight not just a huge burden but also call for more research – use it ☺
  - How generalisable are the results given that it includes only 5 countries from the region. E.g. how different are some regions in Latin America.
  - How much data do Mexico and Brazil contribute to CCASAnet data (not the main contributors when looking at numbers overall but not sure for >50yo). As the previous study into NCD from the region included data from Mexico and Brazil it would be good for authors to clearly highlighted added benefits of this study in discussion in relation to existing evidence (multi-cohort, larger number, more generalisable to whole region etc).
  - Would it be useful to have cohorts with controls as in US and Europe to better understand the excess risk of HIV disease and ART on NCDs

***ANSWER:*** *We appreciate this comment and the reviewer’s suggestions. We did*

*substantial changes to Discussion section to address these points. Please see specifically in the manuscript: pages 17-18, lines 352-364, pages 19, lines 378-381, page 19, lines 384-388, page 19, lines 391-395, pages 19-20, lines 400-402; and page 18, lines 356-360.*

General comments

- Authors could use epidemiological terminology e.g prevalence instead of frequency, proportion etc

***ANSWER:*** *Abstract- Background: lines 42-43, we changed frequency to prevalence and incidence.*

*Introduction: We limited the use of the term frequency. We use it when referring to both measures (prevalence and incidence), and use the specific terms when appropriate (see, for instance, lines 92-93 in page 4, last paragraph of Introduction)*

*Methods: We extended this section and added paragraphs to provide a more detailed description of our analysis. In this section, we specifically define how we use these different measures of frequency. We now explicitly describe how we used estimated prevalence and incidence in the study.*

*Results: We changed the term Proportion for Prevalence in the title and legend of Figure 2. We changed the term Frequency for Prevalence In the title and legend of Table 2.*

- When reporting on which NCDs contribute the largest burden, authors could focus on reporting the 3 main NCDs. The number of ‘main’ NCDs reported varies between abstract, results and discussion.

***ANSWER:*** *We thank the reviewer for pointing these inconsistencies. We reviewed all the manuscript and modified the text in the Abstract and Results sections to make sure the information is consistent along the manuscript.*

- For figures authors may consider using increasingly darker shades from 0 to multimorbidity instead of a darker colour in the middle for 1NCD? If staying in grey shades and increasing in darkness makes it tricky to read consider using patterns (e.g. no pattern=0 NCDs, light pattern is 1 and dark pattern is 2+)

***ANSWER:*** *We followed the reviewer´s advice and modify figures as suggested. In Figure 1, we also added a table documenting the number of patients >50yo receiving care each year.*

Minor comments

- In abstract could authors be more explicit in abstract that NCD prevalence was estimated amongst PLHIV (not general population attending centres).

***ANSWER:*** *We added wording in the abstract to make clear that we studied only people receiving care for HIV and not the general population. This sentence in the Background of the abstract now is read as follows: “We estimated the prevalence and incidence of non-communicable diseases (NCD), including multimorbidity, among people 50 years of age or older (≥50yo) receiving HIV care during 2000-2015 in six centers affiliated with the Caribbean, Central and South American network for HIV epidemiology (CCASAnet****).”*** *(Please see page 2, lines 42-45)*

- In conclusions of abstract authors may also like to elude to the fact that the burden seems to be increasing over time

***ANSWER:*** *We appreciate this comment. In the new version of the manuscript we stated: “The prevalence of NCDs and multimorbidity in people ≥50 years receiving care for HIV in CCASAnet centers in Latin America increased substantially in the last 15 years.” (Please see, pages 2-3, lines 58-60)*

- In introduction could authors state that the study was “from Name et al” instead of “from our colleagues” colleagues?

***ANSWER:*** *We changed this section of the main text as suggested by the reviewer. The new version (Page 3, Lines 75-78) says: “A recent study by Carraquiry et al in our cohort suggested that those starting cART at 50 years or older had an increased risk of death independent of CD4 count or AIDS when compared with people starting cART at younger ages, despite decreased risk of virological failure and treatment modification [9]”.*

- In introduction when reporting on reasons for excess mortality authors should say “possible reason could be the increased presence” instead of “is”

***ANSWER:*** *We changed the text as suggested.*

- Discussion could benefit from use of paragraph to distinguish individual sections.

***ANSWER:*** *We added subheadings to paragraphs in the Discussion section to help distinguish individual sections and better organize this section.*

- In discussion authors state that HTN, dyslipidaemia, psychiatric disorders AND diabetes are the main NCDs but diabetes was not mentioned in result or abstract.

***ANSWER:*** *We reviewed and modified the text in the Abstract and Results sections to make sure the information is consistent along the manuscript.*

- It would strengthen discussion to mention what drives observed trends when discussion difference in NCD burden at enrolment and end of follow-up (higher incidence)

***ANSWER:*** *We discussed this, specifically in the second paragraph of the Discussion section (Page 16, lines 326-331), that says: “We observed a lower prevalence of NCDs at the start of follow-up in patients ≥50yo at enrollment, but at the end of follow-up the prevalence of any comorbidity and multi-morbidity was similar regardless of age at enrollment. While people enrolled in HIV care at 50yo or older had an apparently lower prevalence of NCDs at baseline and higher incidence during follow-up, increased opportunities for diagnosis after enrollment in regular medical care for HIV-infection are likely to account for most of this difference”.*

*We also added in a brief statement, about the potential effect of ART over serum lipids, and how this might have contributed to our findings* *(Pages 16-17 , lines 331-336 )* ***“****Antiretroviral initiation-associated dyslipidemia might also partially explain these differences, as almost all patients in our centers were initiated on efavirenz- or boosted lopinavir-based regimens with ziduvudine and lamivudine during the study period [20, 22]; and dyslipidemia, a common complication of former first-line ART regimes [30], was the most commonly registered incident NCD among patients ≥50yo at enrollment*”

- I am not sure I understand the sentence “Another potential limitation is that the prevalence …”

***ANSWER:*** *We were trying to acknowledge that prevalence of NCDs and multimorbidity are parameters that provide general information about health and need of services for this population, but not a precise indicator of specific healthcare needs in this group. Comprehensive healthcare for aging adults takes into consideration other variables that might be more relevant such as disability, polypharmacy, neurocognitive decline and other geriatric syndromes. We modified the text accordingly, and hope this change contributes to clarify this point (Please see, page 19, lines 378-381).*

- The sentence “As such, we focus… “is misleading given the point on underdiagnosis. Authors may want instead to comment on gaps could be addressed for future analysis (analysis of screening practice to see if it is true underdiagnosis, that underscreening compared to guidelines has also been reported in HIC eg <https://www.ncbi.nlm.nih.gov/pubmed/28578613>.

***ANSWER:*** *We agree and modified this part of the Discussion section as suggested by the reviewer (Page 19, lines 384-391). In the new version, we deleted this sentence and included this: “Despite systematic efforts to improve data quality and management in CCASAnet [25,26], this data is subject to potential information biases: NCDs diagnoses were included solely if recorded in the medical record by the provider, and clinical file management practices may have differed by site and over time; and screening for NCDs is not standardized across center, as previously described* [39]*. Health care seeking behavior, also affects recognition and capture of diagnoses for these conditions and this was one of the reasons we analysed separately groups by age at enrollment in care. Thus, underdiagnosis and under-reporting of NCDs might have led us to underestimate the real frequency of these disorders [40-42].”*

**Journal Requirements**

Please ensure that your manuscript meets PLOS ONE's style requirements, including those for file naming. The PLOS ONE style templates can be found at

<http://www.journals.plos.org/plosone/s/file?id=wjVg/PLOSOne_formatting_sample_main_body.pdf> and <http://www.journals.plos.org/plosone/s/file?id=ba62/PLOSOne_formatting_sample_title_authors_affiliations.pdf>

***ANSWER:*** *Our manuscript meets the style requirements.*

1. Thank you for including your ethics statement: "Institutional review board approval was obtained locally for each participating site and for the DCC at Vanderbilt. In each of the sites contributing data to this study, ethical regulations and policies permit retrospective analysis of de-identified clinical data without informed consent when research is approved by an Institutional Review Board or appropriately constituted ethics committee. "
   a. Please amend your current ethics statement to include the full name of the ethics committee/institutional review board(s) that approved your specific study.
   b. Once you have amended this/these statement(s) in the Methods section of the manuscript, please add the same text to the “Ethics Statement” field of the submission form (via “Edit Submission”).
   For additional information about PLOS ONE ethical requirements for human subjects research, please refer to <http://journals.plos.org/plosone/s/submission-guidelines#loc-human-subjects-research>

***ANSWER:*** *Please see the ethics statement in the methods section which includes the names of each ethics committee.*

“Institutional review board approval was obtained locally for each participating site and for the DCC at Vanderbilt. In each of the sites contributing data to this study, ethical regulations and policies permit retrospective analysis of de-identified clinical data without informed consent when research is approved by an Institutional Review Board or appropriately constituted ethics committee. Argentina – Comité de Bioética de Fundación Huésped. Brazil- Comité de Ética em Pesquisa (CEP), Fundacao Oswaldo Cruz, Instituto Nacional de Infectologia Evandro Chagas. Comissao Nacional de Etica em Pesquisa (CONEP). Chile- Comite Etico Científico del Servicio de Salud Metropolitano Central. Haiti- Comité des Droits Humains des Centres GHESKIO, and Weill Cornel Medical College Institutional Review Board .Honduras –Comité de Ética en Investigación Biomédica de la Unidad de Investigación Científica de la Universidad Nacional autónoma de Honduras. México – Comité de Ética en Investigación del Instituto Nacional de Ciencias Médicas y Nutrición Salvador Zubirán. Perú– Comité Institucional de Ética, Universidad Peruana Cayetano Heredia. VUMC – Vanderbilt University Institutional Review Board.”

2. Thank you for including your competing interests statement; "Pablo F Belaunzaran-Zamudio, Yanink Caro-Vega, Mark J Giganti, Brenda Crabtree-Ramírez, Bryan E Shepherd, Carina Cesar, Rodrigo C Moreira, Fernando Mejia, Marcelo Wolff, Jean W. Pape, Denis Padgett and Catherine C McGowan have no conflicts to declare. Juan Sierra-Madero reports personal fees and non-financial support from Gilead, non-financial support from MSD, grants from BMS, grants from Pfizer, and personal fees from Jansen, all outside the submitted work. "

Please confirm that this does not alter your adherence to all PLOS ONE policies on sharing data and materials, by including the following statement: "This does not alter our adherence to  PLOS ONE policies on sharing data and materials.” (as detailed online in our guide for authors <http://journals.plos.org/plosone/s/competing-interests>).  If there are restrictions on sharing of data and/or materials, please state these. Please note that we cannot proceed with consideration of your article until this information has been declared.

***ANSWER:*** *The competing interests* *statement below includes now some sentences explaining the sharing data principles for CCASAnet as you suggested:*

"Pablo F Belaunzaran-Zamudio, Yanink Caro-Vega, Mark J Giganti, Brenda Crabtree-Ramírez, Bryan E Shepherd, Carina Cesar, Rodrigo C Moreira, Fernando Mejia, Marcelo Wolff, Jean W. Pape, Denis Padgett and Catherine C McGowan have no conflicts to declare. Juan Sierra-Madero reports personal fees and non-financial support from Gilead, non-financial support from MSD, grants from BMS, grants from Pfizer, and personal fees from Jansen, all outside the submitted work. This does not alter our adherence to  PLOS ONE policies on sharing data and materials. Complete data for this study cannot be publicly shared because of legal and ethical restrictions. The Principles of Collaboration under which the CCASAnet multi-national collaboration was founded and the regulatory requirements of the different countries' IRBs require the submission and approval of a project concept sheet by the CCASAnet Executive Committee and the principal investigators at participating sites. All datasets provided by CCASAnet are de-identified according to HIPAA Safe Harbor guidelines. Since re-identification of de-identified datasets may be possible when they are combined with publicly available datasets, CCASAnet promotes the signing of a Data Use Agreement before HIV clinical data can be released. Instructions for how to obtain CCASAnet data are outlined on the CCASAnet website: <https://www.ccasanet.org/collaborate/>. "

Please include your updated Competing Interests statement in your cover letter; we will change the online submission form on your behalf.

Please know it is PLOS ONE policy for corresponding authors to declare, on behalf of all authors, all potential competing interests for the purposes of transparency. PLOS defines a competing interest as anything that interferes with, or could reasonably be perceived as interfering with, the full and objective presentation, peer review, editorial decision-making, or publication of research or non-research articles submitted to one of the journals. Competing interests can be financial or non-financial, professional, or personal. Competing interests can arise in relationship to an organization or another person. Please follow this link to our website for more details on competing interests: <http://journals.plos.org/plosone/s/competing-interests>

*Best Regards,*

***Yanink Caro-Vega***

*Unidad del Paciente Ambulatorio (UPA), 5to piso*

*Vasco de Quiroga # 15
Col. Sección XVI
Delegación Tlalpan; C.P. 14000
México D.F.; México
Tel. +52 (55)54870900; ext, 5505
Fax. +52 (55)55130010*

*yanink.caro@infecto.mx*
