# Supplementary material for: Frequency of non-communicable diseases in people 50 years of age and older receiving HIV care in Latin America
Source: PLoS One. 2020 Jun 17;15(6):e0233965. doi: 10.1371/journal.pone.0233965 (PMC7299309; doi:10.1371/journal.pone.0233965)
Supplement: Attachment — Submitted filename: 2_response_to_rev_1.2_20200507.docx [file pone.0233965.s003.docx]

**Response to Reviewers**

Dear Dr. Pereira:

We appreciate the interest of PLoS ONE in our manuscript and welcome the feedback of a new reviewer. We think these comments helped us to improve our manuscript and contributed to convey our ideas clearer in the new draft we are submitting for your consideration. Next, we respond to each point raised by the new reviewer, addressing the changes each comment lead to in the new version. In addition to the specific changes listed below, we reorganized a subheading of the *Discussion* section (*Limitations)* to better organize the changes and for clarity purposes. All changes were tracked using the Words function.

**Comments by Reviewer #3:**

The report of the Frequency of non-communicable diseases in people 50 years of age and older receiving HIV care in Latin America by Belaunzaran-Zamudio and colleagues presents information about the frequency of non-communicable diseases (NCDs) in persons receiving treatment for HIV related conditions; an area where there is limited data in Latin America and the Caribbean.

However, in my opinion, there are several elements of this paper that still require justification and/or further clarification.

***Comment:*** The authors note the high prevalences of NCDs in single center cohorts of older persons being treated for HIV in Mexico and Brazil (lines 83-86), but do not describe whether these rates differ from comparable rates in groups without HIV.

***Author´s response:*** In the revised manuscript, we have included some published data on NCDs among persons without HIV. We used published information from age-specific, population-based estimates from the Health and Nutrition National Survey in Mexico, the Telephonic Survey for Health Risk Surveillance in Brazil, a population-based study in rural, western Honduras, and a national urban population-based longitudinal study in Peru (PERUDIAB). These data provide some context for our analysis. In these surveys, the prevalence of a few NCDs in people of 50 years of age and older is actually high. We added the next sentences in the third paragraph (*page 4, lines 81-88*) of the *Introduction* section (and added the citations for new references used to support the statement):

*“Among the general population of people aged 50 or older in Latin America, the prevalence of NCDs is high. For example, recent studies from Brazil [11,12], Honduras [13], Mexico [14-16], and Peru [17] have all reported estimates of the prevalence of diabetes mellitus (DM) in older populations to be 12% or higher. Among those between the ages of 60 and 70 in Mexico, the estimated prevalence was as high as 36% [14]. The percentage of people 50 years and older with hypertension (HTN) was estimated to be greater than 40% in urban and rural settings in Mexico [14-16], Brazil [11,12], and Honduras [13]. Dyslipidemia was also higher than 35% in both Brazil and Mexico [11,12,16].”*

There are of course some challenges to comparing our estimates with those from the general populations. In particular, the methodologies for collecting the information are different, ascertainment may vary, and there could be discrepancies/bias in who is included in the estimates. We also modified the *Discussion* section to elaborate on possible explanations for an overall lower prevalence of some of these NCDs than in the general population in our region. Thus, we added a paragraph in the subheading *Results of the study in context (page 18, lines 380-384):*

*“We observed a lower prevalence of NCDs in our cohort relative to prevalence estimates corresponding to the general population in Latin America. [11-17]. It is unclear whether this is due to incomplete ascertainment of NCDs or selection biases that make a cohort of patients longitudinally receiving treatment and care at a health center not comparable with a representative sample of the general population.”*

***Comment:*** A major concern of this paper is the methodology used in the analysis as the authors do not provide satisfactory justification for dichotomizing the study sample. They state; ‘and in our own exploratory data analysis, people enrolled in care at older age appear to be fundamentally different than people that enrolled younger and aged receiving care and ART’ [lines 164 – 166]. As such the authors should present supporting data to support this view and to justify the methods that they used. They cite two references by Guardali et al (references 11 and 18). When one evaluates the BMC Geriatrics reference (11), the factor which is associated with NCD risk in persons with HIV is not age, but rather duration of HIV treatment.

***Author´s response:*** We appreciate this comment by the reviewer, as it allowed us to better contextualize our work. In this study, we build-up from our previous research (Caro-Vega Y, *et al*. Epidemiol Infect. 2018) where we described how the percentage of patients over 50 years increased from 8% to 24% from 2000 to 2015. During this period, people enrolled younger in care and receiving ART contribute differently to the older HIV population growth than those enrolled at older ages. We decided to maintain this categorization to explore how much each group might have contributed to the prevalence of comorbidities and multi-morbidity in our region. To clarify this point, we modified the *Methods* section with the following sentences (*page 7, lines 164-170*, *subheading Study population*)

*“While the classification of ‘older PLWHIV” is not explicitly defined, a threshold of 50 years of age is commonly used to indicate an aging or older HIV population [35]. In our own research, we have used this threshold to assess how people enrolled at younger ages and receiving ART have contributed to growth of the older PLWHIV population compared to those enrolled at older ages [7]. We maintain this categorization to explore how much each group might contribute to the prevalence of comorbidities and multi-morbidity in our region.”*

The results presented in the studies by Guaraldi and collaborators (Guaraldi et al, BMC Geriatrics 2018; Guaraldi et al. PLoS ONE 2015) shows that both, age and time since diagnosis of HIV (not exactly time on ART, which was not measured but which is likely positively correlated with time since diagnosis) are associated with some comorbidities, multimorbidity and polypharmacy, as can be inferred from results summarized in tables 1 & 2, and figures 2 & 4 of that manuscript.

These results support our decision to categorize our study population in those that enrolled in care before versus after 50 years of age, as those diagnosed and enrolled in care at younger ages have been enrolled in care longer, exposed to ART longer, and probably have been HIV-infected longer than those enrolled in care at older ages. We provide this as a rationale for our decision (*see page 4 & 5, lines 102-105*).

*“Patients were stratified by age at enrollment in care (<50 years at enrollment and ≥ 50 years at enrollment) given possible differences in time of ART exposure, previous health history, HIV duration and severity, clinical outcomes, and mortality between people aging in care and people diagnosed at older age [18, 26].”*

Furthermore, the results presented in Table 1 support our view, and make evident that not only the mean age of these groups is quantitatively different but crucially, people enrolled in care at older ages have different time in follow-up than those enrolled younger and started receiving care more recently. Our results and conclusions are overall in agreement with those by Guaraldi in that manuscript.

In response to the reviewers´ comments, we added some additional information relevant to this subject. We modified the text in the *Methods* section to better explain our rationale for this decision (*page 7*, *lines 164-170*). We also added 2 rows In Table 1 summarizing the information about the frequency of ART use and time on ART in each group. We mentioned these differences in the *Results,* *page 11 (lines 240-243).* This information strengthens our rationale for categorizing this population based in age at enrollment in care.

In the modified text in the Methods section, we now state the following:

*“While the classification of ‘older PLWHIV” is not explicitly defined, a threshold of 50 years of age is commonly used to indicate an aging or older HIV population [35]. In our own research, we have used this threshold to assess how people enrolled at younger ages and receiving ART have contributed to growth of the older PLWHIV population compared to those enrolled at older ages [7]. We maintain this categorization to explore how much each group might contribute to the prevalence of comorbidities and multi-morbidity in our region.”*

In the modified text in the Results section, we now state the following:

*“Patients <50yo at enrollment were followed a median time of 3.1 years after reaching 50 years (cumulative time of 7,821 years in follow-up). In contrast, those ≥50yo at enrollment were followed for 4.6 years (8,064 cumulative years in follow-up).”*

We agree with the reviewer that this approach has limitations that need to be discussed in our manuscript. In the new draft, we discuss the limitations and advantages of our approach in the context of previously published literature (*see pages 19-20, lines 415-420*), where we state the following:

*“We classified our study population in two groups based on their age at enrollment in care. We acknowledge this is an arbitrary distinction that does not necessarily accurately depict a complex aging biological process intertwined with the effects of long-term toxicity of ART. Nonetheless, ours is a descriptive study aiming to quantify this emerging problem and we found this a useful solution to describe separately subgroups in our population of interest that present different morbidity patterns.”*

***Comment:*** This study is presents as both a prevalence study and a cohort study where incident outcomes are reported. A concern is the very short median period of follow up of only 3.7 years at ages ≥50 years [233]. Given that over the 15-year period of review, persons would have died, been lost to follow up or excluded for reasons such as non-attendance, in the absence of more comprehensive data regarding participation by study subjects, it is difficult to understand actual participation by study subjects or potential biases that might have resulted from non-participation/losses to follow up, or indeed patterns of participation.

***Author´s response:*** Patients <50yo at enrollment were followed for a cumulative time of 7,821 years; and those ≥50yo at enrollment were followed for 8,064 cumulative years We do not consider a median follow-up time of almost 4 years as short.

While the study period encompasses 15 years of enrollment and follow-up, 50% of enrollment in this cohort occurred between 2010 and 2015 (see Table 1). Most patients (77%) were followed-up until 2015 and administratively censored (79% in patients enrolled<=50 and 73% in those enrolled ≥50yo), 15% were lost to follow-up (LTFU) in both groups, and 8% deaths (5% in patients enrolled<=50 and 11% in those enrolled ≥50yo). We included this information in the *Results* section of new draft of the manuscript (*see Page 11, lines 246-253*).

“*While the study period encompasses a 15-year period (2000-2015), 50% of all person time of observation in this study occurred between 2010 and 2015 (Table 1). Most patients were followed-up until 2015 and were administratively censored (79% in patients enrolled<=50 and 73% in those enrolled ≥50yo). The remaining participants were lost to follow-up (17%) or died (8%). Compared to those ≥50yo at enrollment, a higher proportion of patients <50yo at enrollment were administratively censored (79% vs 73%, respectively) and a lower proportion died (5% vs 11%, respectively).”*

***Comment:*** Additionally, regarding reported prevalence data, there is the possibility of biases linked to differences in survival or participation for those without NCDs compared to those with NCDs. The authors note that ‘We observed a lower prevalence of NCDs at the start of follow-up in patients ≥50yo at enrollment, [line 327]. Advancing this argument, then the reported increased prevalence of NCDs over time, might have been a feature of better access to improved care associated with increased survival, but greater multimorbidity. This is relevant as participating subjects repeatedly contribute to annual prevalence data.

***Author´s response:*** We acknowledge this observation is likely the result of better access to improved care at different time in for each age-group. Please see discussion section, at “Frequency of NCDs” subsection, *page 16,* *lines 344-347*:

“*While people enrolled in HIV care at 50yo or older had an apparently lower prevalence of NCDs at baseline and higher incidence during follow-up, increased opportunities for diagnosis after enrollment in regular medical care for HIV-infection are likely to account for most of this difference*.”

***Comment:*** The observation regarding the lower occurrence of NCDs in this cohort of persons with HIV in Latin America compared to higher income countries [lines 342-346] clearly is the result of several factors which might include differences in participation in care, quality of care, study methodological issues (sampling, biases etc.. ). It would be important and relevant for the authors to evaluate whether the burden of NCDs is in fact different in this population of persons being treated for HIV compared to similar unaffected populations in the Region.

***Author´s response:*** The purpose of this study is to quantify the frequency of NCDs in this population, which to date has been understudied in our region. We added text regarding the frequency of NCDs among older people in the general population in Latin America, to provide more background about the burden of these disorders in the general population in Latin America.

Please see introduction, *page 4,* *lines 81-88*:

*“Among the general population of people aged 50 or older in Latin America, the prevalence of NCDs is high. For example, recent studies from Brazil [11,12], Honduras [13], Mexico [14-16], and Peru [17] have all reported estimates of the prevalence of diabetes mellitus (DM) in older populations to be 12% or higher. Among those between the ages of 60 and 70 in Mexico, the estimated prevalence was as high as 36% [14]. The percentage of people 50 years and older with hypertension (HTN) was estimated to be greater than 40% in urban and rural settings in Mexico [14-16], Brazil [11,12], and Honduras [13]. Dyslipidemia was also higher than 35% in both Brazil and Mexico [11,12,16].”*

We also elaborate in the discussion section, please see *page 18* *lines 380-384:*

*“We observed a lower prevalence of NCDs in our cohort relative to prevalence estimates corresponding to the general population in Latin America. [11-17]. It is unclear whether this is due to incomplete ascertainment of NCDs or selection biases that make a cohort of patients longitudinally receiving treatment and care at a health center not comparable with a representative sample of the general population.”*

We also acknowledge that differences with previous studies in high-income countries are likely to be multifactorial, including characteristics of the structure of the cohort (please see *pages 17-18, lines 360-371*):

*“Differences in the frequency of environmental, genetic and individual risk factors for NCDs might explain these disparities [41,42].Previous studies in Italy found that people aging while receiving HIV care more frequently had several specific NCDs, multimorbidity, and polypharmacy than people of similar age diagnosed and enrolled in care later in life [18,26]. In contrast, we saw that patients enrolled in care after 50 years of age had a lower prevalence of NCDs than those who reached 50 years of age while in care, whereas those enrolled after 50 years had a higher incidence of NCDs. This discrepancy may potentially be explained by the shorter time in observation for both groups in our study and considerable larger differences of time living with HIV between groups in Italy (≥10 years in comparison with around 5 years in this study), rather than age itself [18,22,26].”*

7. PLOS authors have the option to publish the peer review history of their article ([what does this mean?](https://journals.plos.org/plosone/s/editorial-and-peer-review-process#loc-peer-review-history)). If published, this will include your full peer review and any attached files.

If you choose “no”, your identity will remain anonymous but your review may still be made public.

**Do you want your identity to be public for this peer review?** For information about this choice, including consent withdrawal, please see our [Privacy Policy](https://www.plos.org/privacy-policy).

Reviewer #3: No
